# A retrospective study of locally advanced cervical cancer cases treated with CT-based 3D-IGBT compared with 2D-IGBT

**DOI:** 10.1007/s11604-023-01439-6

**Published:** 2023-05-04

**Authors:** Toshifumi Kinoshita, Shigeo Takahashi, Masahide Anada, Takamasa Nishide, Kenji Kanenishi, Akinori Kawada, Toru Shibata

**Affiliations:** 1 Department of Radiation Oncology, Kagawa University Hospital, 1750-1 Ikenobe, Kita-Gun, Miki-Cho, Kagawa 761-0793 Japan; 2 Department of Radiation Oncology, Kagawa Rosai Hospital, 3-3-1 Joto-Cho, Marugame, Kagawa 763-8502 Japan; 3 Department of Obstetrics and Gynecology, Kagawa Rosai Hospital, 3-3-1 Joto-Cho, Marugame, Kagawa 763-8502 Japan; 4 Department of Perinatology and Gynecology, Kagawa University Hospital, 1750-1 Ikenobe, Kita-Gun, Miki-Cho, Kagawa 761-0793 Japan

**Keywords:** Locally advanced cervical cancer, CT-based 3D-IGBT, Outcome, Bavazcizumab

## Abstract

**Purpose:**

To retrospectively review locally advanced cervical cancer (CC) cases treated with three-dimensional image-guided brachytherapy (3D-IGBT) and two-dimensional (2D)-IGBT.

**Materials and Methods:**

Patients with Stage IB–IVa CC who underwent intracavitary irradiation between 2007 and 2021 were divided into the 3D-IGBT and 2D-IGBT groups. Local control (LC), distant metastasis-free survival (DMFS), progression-free survival (PFS), overall survival (OS), and gastrointestinal toxicity (G3 or more) were investigated at 2/3 years post-treatment.

**Results:**

Seventy-one patients in the 2D-IGBT group from 2007 to 2016 and 61 patients in the 3D-IGBT group from 2016–2021 were included in the study. The median follow-up period was 72.7 (4.6–183.9) months in the 2D-IGBT group and 30.0 (4.2–70.5) months in the 3D-IGBT group. The median age was 65.0 (40–93) years in the 2D-IGBT group and 60.0 (28–87) years in the 3D-IGBT group, but there was no difference in FIGO stage, histology, or tumor size between the groups. In treatment, the median A point dose was 56.1 (40.0–74.0) Gy in the 2D-IGBT group and 64.0 (52.0–76.8) Gy in the 3D-IGBT group (P < 0.0001), and the proportion of patients who underwent chemotherapy more than five times was 54.3% in the 2D-IGBT group and 80.8% in the 3D-IGBT group (P = 0.0004). The 2/3-year LC, DMFS, PFS, and OS rates were 87.3%/85.5%, 77.4%/65.0%, 69.9%/59.9%, and 87.9%/77.9% in the 2D-IGBT group, and 94.2%/94.2%, 81.8%/81.8%, 80.5%/80.5%, and 91.6%/83.0% in the 3D-IGBT group, respectively. A significant difference was observed in PFS (P = 0.02). There was no difference in gastrointestinal toxicity, but there were four intestinal perforations in the patients from the 3D-IGBT group, three of whom had a history of bevacizumab treatment.

**Conclusion:**

The 2/3-year LC of the 3D-IGBT group was excellent and PFS also tended to improve. Care should be taken with concomitant use of bevacizumab after radiotherapy.

## Introduction

Cervical cancer (CC) is one of the most common malignant diseases affecting female individuals, remaining a major cause of cancer death among female adolescents and young adults [1, 2]. The standard treatment for locally advanced cervical cancer (LACC) is concurrent chemoradiotherapy (CCRT) [3–7]. The radiotherapy (RT) aspect of the treatment often comprises external beam radiotherapy (EBRT) and brachytherapy (BT). Recently, the Retro-EMBRACE cohort study reported excellent local control (LC) and pelvic control (91% and 87% at 3 years, respectively) as well as overall survival (OS) and cancer-specific survival rates (74% and 79% at 3 years, respectively) with limited severe morbidity [8]. However, regardless of excellent LC, the high proportion of distant metastasis (DM) remains an important issue related to this disease.

The purpose of this retrospective study was to examine LC, distant metastasis-free survival (DMFS), progression-free survival (PFS), and OS among patients with CC who received definitive RT with BT from two institutions. Moreover, we aimed to compare cases treated with three-dimensional image-guided brachytherapy (3D-IGBT) and two-dimensional (2D)-IGBT. Moreover, we searched for current status and problems related to this treatment.

## Materials and methods

### Patients

The data from consecutive CC patients who received treatment from two institutions between 2007 and 2021 were retrospectively analyzed. This study was approved by the Institutional Review Boards of the involved institutions. We included patients pathologically diagnosed with International Federation of Gynecology and Obstetrics (FIGO) 2008 Stages Ib–IVa who were treated with EBRT followed by high dose–rate intracavitary BT. BT was performed at the same facility. We excluded patients treated with RT without BT. Moreover, the patient cohort included those with para-aortic lymph node metastasis.

### Treatment

In this study, the RT protocol was composed of a combination of EBRT and BT. The first course of whole-pelvic external irradiation was delivered at a dose of 1.8–2.0 Gy/fraction, five times/week, up to a total dose of 20–50.4 Gy. The second course of EBRT was performed with central shielding up to a total external dose of 50–50.4 Gy. Boost EBRT for nodal metastases was delivered using 4–10 Gy in 2–5 fractions. From 2020, EBRT was conducted with intensity-modulated radiotherapy (IMRT) or simultaneous integrated boost (SIB)-IMRT. The doses of SIB-IMRT were 45 Gy/25 fr for regional and 55 Gy/25 fr for lymph node metastasis. BT was performed with 2–4 cycles once or twice a week at a fractional dose of 5.5–6.5 Gy, for a total dose of 12–24 Gy at point A. From 2007 to 2015, BT with 2D-IGBT, was used, while after 2016, computed tomography (CT)-guided 3D-IGBT was used. In our institution, BT was conducted using tandem and ovoid. This therapy was performed through microSelectron v2 (Nucletron, Elekta Company, Stockholm, Sweden). For the methods of contouring of the organs at risk (OARs) and for the high-risk clinical target volume (HR-CTV), we referred to the recommendations from the gynecological (GYN) GEC ESTRO working group [9] and the Working Group of the Gynecological Tumor Committee of the Japanese Radiation Oncology Study Group [10]. The dose per fraction for HR-CTV D90 should be 5.8–6.0 Gy or higher. Those for the OARs were set at ≤ 6 Gy for rectum D2cc and sigmoid colon D2cc and at ≤ 7 Gy for bladder D2cc. The total doses of whole-pelvic irradiation and BT at point A were calculated using a biologically equivalent dose to 2 Gy/fraction (EQD2) through the linear-quadratic model, wherein α/β = 10 Gy. For OARs, the calculation was made with α/β = 3 Gy. The doses from whole-pelvic with central shielding were excluded in the total EQD2. The EQD2 total dose for HR-CTV was set to be ≥ 60 Gy. Those for rectum D2cc and sigmoid colon D2cc were set to be ≤ 75 Gy, while those for bladder D2cc were ≤ 85 Gy. Patients with para-aortic lymph node metastasis received extended radiation treatment.

For chemotherapy (CTx), cisplatin (40 mg/m^2^) or paclitaxel (60 mg/m^2^) plus a platinum antitumor agent (nedaplatin 25 mg/m^2^ or carboplatin with an area under the curve of 2 mg/mL/min) was administered weekly according to institutional policy.

### Outcomes and statistical analyses

Statistical analyses were conducted using R version 3.3.4 (R Foundation for Statistical Computing, Vienna, Austria). Kaplan–Meier analysis was used to estimate LC, DMFS, PFS, and OS. We examined age, stage, histology, pelvic lymph node metastasis, para-aortic lymph node metastasis, tumor size, overall treatment time, point A dose (EQD2), CTx use, and the frequency of CTx. Fischer’s exact test was used to evaluate differences between the 2D-IGBT and 3D-IGBT groups. P values < 0.05 were considered to indicate statistical significance.

To evaluate late toxicity, the National Cancer Institute Common Terminology Criteria for Adverse Events (version 4.0) were used [11].

## Results

### Patient characteristics

A total of 132 patients with CC who received definitive RT were included in this study. A total of 92 cases came from one institution and the remaining 40 came from another. The patient, stage, tumor, and treatment characteristics are shown in Table 1. A total of 71 patients in the 2D-IGBT group from 2007 to 2016 and 61 patients in the 3D-IGBT group from 2016 to 2021 were included in the study. There were two patients treated with combined intracavitary and interstitial brachytherapy in 2021. There was no significant difference in the FIGO stage, histology, tumor size, and presence of lymph node metastasis between the groups. For treatment, there was no difference in the overall treatment time (OTT) between the two groups. The median EQD2 was 56.2 (40.0–74.0) Gy in the 2D-IGBT group and 64.1 (52.0–76.8) Gy in the 3D-IGBT group, and there was a significant difference between the two groups (P < 0.0001). The median dose of HR-CTV D90 was 70.9 (53.7–93.5) Gy in the 3D-IGBT group. The proportion of concurrent chemoradiotherapy (CCRT) was 64% in the 2D-IGBT group and 85% in the 3D-IGBT group. CCRT was common in the 3D-IGBT group. The percentage of patients who received five or more times of CTx was 54.3% in the 2D-IGBT group and 80.8% in the 3D-IGBT group (P = 0.0004).

**Table 1:** Patient, tumor, and treatment characteristics

	2D-IGBT^a^ (2007–2016)	3D-IGBT^b^ (2017–2021)	P value
Number of cases	71	61	
Follow up period (months)			
Median (range)	72.7 (4.6–183.9)	30.0 (4.2–70.5)	< 0.0001
Age (years)	65.0 (40–93)	60.0 (28–87)	0.0125
Stage			
Ib/II/III/IVa	7/28/29/7	6/25/21/9	0.7998
Histology			
Squamous ca.^c^/Adeno.ca.^d^/Other	59/10/2	59/6/0	0.4765
Tumor diameter^e^ (cm)	5.0 (1–10)	5.7 (1–8.6)	0.1468
Pelvic lymph node involvement^f^			
Yes/No	35/36	41/20	0.0518
Para-aortic lymph node involvement			
Yes/No	11/60	14/47	0.3732
Pretreatment hemoglobin level(g/dL)	11.1 (7.3–14.8)	12.0 (6.4–15.0)	0.1098
Overall treatment time (days)	48 (36–85)	48 (38–63)	0.3409
Point A dose (Gy^g^)	56.1 (40–74)	64.0 (52–76.8)	< 0.0001
HR-CTV^h^ D90 (Gy^g^)		70.9 (53.7–93.5)	
Bladder D2cc (Gy^g^)		77.6 (64.1–105.2)	
Rectum D2cc (Gy^g^)		58.5 (39.6–58.5)	
Sigmoid colon D2cc (Gy^g^)		58.2 (35.7–73.8)	
Chemotherapy (CTx)			
Yes/No	46/25	52/9	0.0093
Times of CTx			
Less than five times/Five times or more	21/25	10/42	0.0004
Adjuvant CTx			
Yes/No	40/31	32/29	0.7268
Local recurrence (cases)	10	3	
Regional recurrence^i^ (cases)	8	4	
Distant metastasis^j^ (cases)	28	10	
All recurrence (cases)	32	11	

^a^Brachytherapy with two-dimensional imaging

^b^Brachytherapy with CT-guided three-dimensional imaging

^c^Squamous cell carcinoma

^d^Adenocarcinoma

^e^Tumor diameter was measured using the maximum length meter

^f^Lymph node involvement was defined as a diameter > 1 cm or positive FDG-PET

^g^EQD2 (biologically equivalent dose; reference dose per fraction = 2 Gy, linear quadratic model, α/β = 10 Gy point A and HR-CTV, α/β = 3 Gy Bladder, Rectum, and Sigmoid colon)

^h^High-risk clinical target volume

^i^Regional recurrence was defined as recurrence inside the radiation field except for the cervix of the uterus

^j^Distant metastasis was defined as recurrence outside the radiation field

### Tumor response

There were 32 relapsed cases in the 2D-IGBT group and 11 in the 3D-IGBT group. Eight out of 11 cases in the 3D-IGBT group were treated with paclitaxel plus platinum-based antitumor agents, four with irinotecan, and five with bevacizumab. Bevacizumab was used in combination.

Local recurrence occurred in 13 patients, 10 of whom were in the 2D-IGBT group and three were in the 3D-IGBT group. One case had local recurrence only and all the other cases had metastases. The patient with local recurrence only refused treatment and was transferred to another hospital. Of the 12 patients with metastases, one patient underwent hysterectomy and CTx, and one patient received additional radiation therapy. Two patients with additional local treatment died of metastases in other sites. The other seven patients received CTx only, and three could not be treated due to unfavorable conditions. Regional recurrence occurred in 12 patients, eight of whom were in the 2D-IGBT group and four were in the 3D-IGBT group. Distant metastasis occurred in 38 patients, 28 of whom were in the 2D-IGBT group and 10 were in the 3D-IGBT group.

**Fig. 1:**
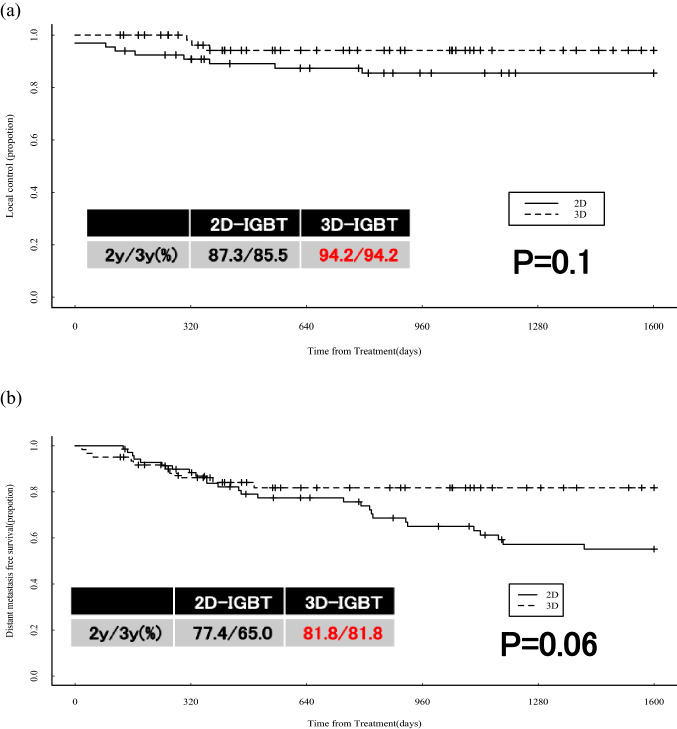

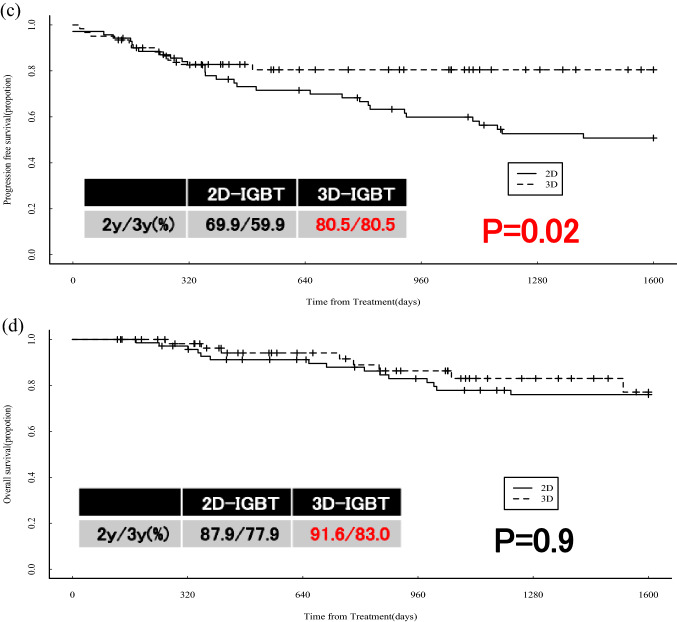
Patient outcomes: **a** local control, **b** distant-metastasis free survival, **c** progression-free survival, and **d** overall survival

The results of the statistical analysis are shown in Fig. 1a–d. The 2/3 year LC, DMFS, PFS, and OS rates were 87.3%/85.5%, 77.4%/65.0%, 69.9%/59.9%, and 87.9%/77.9% in the 2D-IGBT group and 94.2%/94.2%, 81.8%/81.8%, 80.5%/80.5%, and 91.6%/83.0% in the 3D-IGBT group, respectively. LC and DMFS tended to improve in the 3D-IGBT group, and PFS was significantly better in the 3D-IGBT group than in the 2D-IGBT group (P = 0.02). There was no significant difference between the two groups for OS.

### Late toxicity

**Fig. 2:**
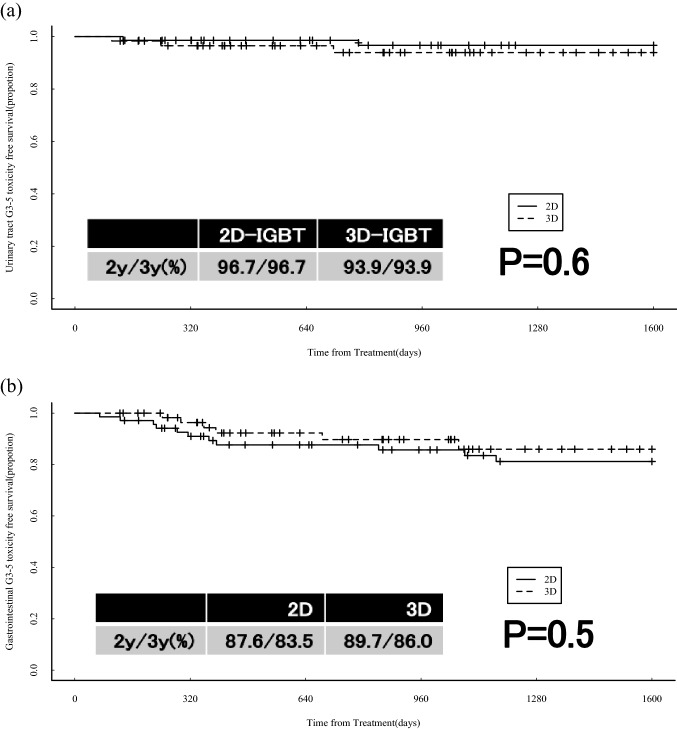
Comparison of the rates of grade 3 or more adverse events between the 2D-IGBT and 3D-IGBT groups. **a** G3-5 urinary tract adverse events free survival, **b** G3-gastrointestinal adverse events free survival

The rates of grade 3 (G3) or greater urinary tract and gastrointestinal (GI) adverse events (AE) were 3.3%/3.3% and 12.4%/16.5% in the 2D-IGBT group and 6.1%/6.1% and 10.3%/14.0% in the 3D-IGBT group after 2 and 3 years of follow-up, respectively. The results are shown in Fig. 2a, b; there was no significant difference between the two groups. The complication details are presented in Table 2a, b. There were two cases of bladder perforation in each of the two groups. All patients with fistula were not associated with bladder invasion. In the 2D-IGBT group, one patient underwent neoadjuvant CTx and the other underwent hysterectomy within 3 months after CCRT. In the 3D-IGBT group, one patient underwent treatment with bevacizumab and the other had related regional recurrence. There were four cases of intestinal perforation in each of the two groups. Three of the four patients in the 3D-IGBT group had previously been treated with bevacizumab. Eleven cases of recurrence occurred in the 3D-IGBT group, and intestinal perforation occurred in one out of six cases, which did not receive bevacizumab, and in three out of five cases of patients receiving bevacizumab (Table 3a, b).

**Table 2:** Adverse events ≥ G3

Adverse Events ≥ G3	2D-IGBT (n = 71)	3D-IGBT (n = 61)
a. Urinary tract		
Bleeding	2	0
Stenosis	0	1
Fistula	2	2
Total	4	3
b. Gastrointestinal tract		
Bleeding	4	2
Ileus	3	0
Stenosis	2	0
Fistula	4	4
Total	13	6

**Table 3:** Perforation with or without bevacizumab in the 3D-IGBT group

Recurrence in the 3D-IGBT group	Bladder perforation (No)	Bladder perforation (Yes)	Total
a. Urinary tract			
With bevacizumab	4	1	5
Without bevacizumab	5	1	6
Total	9	2	11

Recurrence in the 3D-IGBT group	Intestinal perforation (No)	Intestinal perforation (Yes)	Total
b. Gastrointestinal tract			
With bevacizumab	2	3	5
Without bevacizumab	5	1	6
Total	7	4	11

### IMRT

Eight patients were treated with IMRT or SIB-IMRT. Especially, seven of the eight patients were of Stage IIIb while the other patient was of Stage IIb. We confirmed the presence of uterine and cervical tumors within the planning target volume by daily cone beam CT. No adverse effects of acute gastroenteritis above G3, such as diarrhea, were observed. Although the follow-up period was short, as it started in 2020, no bowel perforation was observed.

## Discussion

In this study, we confirmed that the radiation dose can be escalated and that it shows good LC with 3D-IGBT. The 2/3-year LC rate was 94.2%/94.2% in the 3D-IGBT group. The previous study in RetroEMBRACE, a multicenter cohort study, reported that the overall 3/5 year LC rate was 91%/89% [8]. Our results were consistent, although it was still a short follow-up period. The global standard CTV-HR prescription dose is > 85 Gy [12], but the prescription dose in this study was low. Previous studies by Toita et al. [13, 14] have demonstrated that CCRT with a low cumulative RT dose schedule achieved comparable outcomes to global dose schedules. Our study also included cases treated with doses similar to those reported by Toita et al. If necessary, we referred patients to receive a post-treatment diffusion-weighted magnetic resonance imaging (DW-MRI) and gynecological examination for additional BT.

Evaluation of local recurrence immediately after RT can sometimes be difficult. Kalash et al. reported that patients at risk for persistent disease may be accurately diagnosed by DW-MRI and positron emission tomography/computed tomography [15]. Several studies have demonstrated the usefulness of the apparent diffusion coefficient in DW-MRI [16, 17].

A Cochrane meta-analysis of several trials performed showed that CCRT was associated with a 6% improvement in 5-year survival compared with RT alone [18]. However, these groups also reported that more advanced stages had less of a CTx benefit. In contrast, according to a randomized clinical trial by Shrivastava et al., CCRT using weekly cisplatin compared with definitive RT alone still elicited a beneficial response among patients with LACC with Stage IIIb, with an absolute benefit of 8.5% in DFS and 8% in OS [19]. A previous study by Schmid et al. also reported that decreasing the number of cycles of cisplatin may increase DM in patients with Stage IIIb–IVa tumors or positive lymph nodes [20] and that the frequency of CTx during RT was considered to be the most important factor for DM. In our study, the cases in the 3D-IGBT group received a greater number of CTx treatments. Although the difference was not significant, we observed improved DMFS in the 3D-IGBT group over the 2D-IGBT group. PFS was significantly better in the 3D-IGBT group due to improved LC and DMFS.

There was a relatively higher incidence of GI tract AEs > G3. Reduction in this number is expected by 3D-IGBT in the future, as reported by Charra–Brunaud et al. [21]. However, in our study, the 2/3 year rates of GI tract AEs > G3 were 12.4%/16.5% in the 2D-IGBT group and 10.3%/14.0% in the 3D-IGBT group, respectively. GI toxicity was not reduced, even in the 3D-IGBT group. Four patients in the 3D-IGBT group had intestinal perforation, three of whom had been previously treated with bevacizumab. In recent years, the usefulness of bevacizumab for advanced cervical cancer and its combination with an immune checkpoint inhibitor has also been reported [22, 23], and a further increase in treatment opportunities is expected. In the future, caution should be exercised with this combination therapy.

In some cases, much better outcomes may be obtained by treatment with IMRT, as reported by Gandhi et al. [24] and Yeung et al. [25]. We would like to use modern techniques, such as IMRT, to further reduce side effects. Currently, we have started curative treatment of cervical cancer using IMRT and 3D-IGBT as a prospective study. By lowering the dose in the OARs, we aim to reduce the development of severe late adverse effects.

## Conclusion

The 2/3-year LC of the 3D-IGBT group was excellent and PFS also tended to improve. Care should be taken with the concomitant use of bevacizumab after radiotherapy.
